# Supplementary material for: Dietary Diversity and Dietary Patterns in School-Aged Children in Western Kenya: A Latent Class Analysis
Source: Int J Environ Res Public Health. 2022 Jul 26;19(15):9130. doi: 10.3390/ijerph19159130 (PMC9368572; doi:10.3390/ijerph19159130)
Supplement: Supplementary file 1 [file ijerph-19-09130-s001.zip › ijerph-1831096-supplementary.pdf]

Dietary Diversity and Dietary Patterns in School-Aged Children in Western Kenya: A Latent Class Analysis

Tiange Liu,[1*] Sherryl Broverman,[2,3] Eve S. Puffer,[3,4] Daniel Zaltz,[1] Andrew L. Thorne-Lyman,[5] Sara E. Benjamin-Neelon.[1,5]

[1] Department of Health, Behavior and Society, Johns Hopkins Bloomberg School of Public Health, Baltimore, Maryland, 21205, USA

[2] Department of Biology, Duke University, Durham, North Carolina, 27708, USA

[3] Duke Global Health Institute, Duke University Medical Center, Durham, 27710, North Carolina, USA

[4] Department of Psychology and Neuroscience, Duke University, Durham, 27708, North Carolina, USA

[5] Center for Human Nutrition, Department of International Health, Johns Hopkins Bloomberg School of Public Health, Baltimore, Maryland, 21205, USA

*** Corresponding author:**

Tiange Liu

615 N. Wolfe Street, Baltimore, Maryland 21205

email: tliu57@jhu.edu

SUPPLEMENTARY MATERIALS

Table S1. Food groups and cut points for low, medium, and high consumption

	Consumption (g)					
	Village A			Village B		
**Food Group**	Low	Medium	High	Low	Medium	High
1) Grains, white roots and tubers, and plantains	≤220.3	>220.3 to ≤489.1	>489.1	≤63.4	>63.4 to ≤300.5	>300.5
2) Pulses (beans, peas and lentils)	0.0	>0.0	N/A[a]	0.0	>0.0 to ≤28.0	>28.0
3) Nuts and seeds	0.0	>0.0	N/A[a]	0.0	>0.0	N/A[a]
4) Dairy	0.0	>0.0 to ≤6.8	>6.8	0.0	>0.0	N/A[a]
5) Meat, poultry and fish	0.0	>0.0 to ≤79.4	>79.4	≤10.6	>10.6 to ≤87.2	>87.2
6) Eggs	0.0	>0.0	N/A[a]	0.0	>0.0	N/A[a]
7) Dark green leafy vegetables	≤10.1	>10.01 to ≤102.1	>102.1	0.0	>0.0 to ≤51.1	>51.1
8) Other Vitamin A-rich fruits and vegetables	0.0	>0.0	N/A[a]	0.0	>0.0	N/A[a]
9) Other vegetables	≤6.7	>6.7 to ≤25.3	>25.3	≤1.47	>1.47 to ≤11.1	>11.1
10) Other fruits	0.0	>0.0 to ≤175.0	>175.0	0.0	>0.0	N/A[a]

[a]Cut points for low, medium, and high consumption were at the 20th and 80th percentile of each food group, separately for children in village A and B. Some of the food groups had only two categories (0 vs. >0) because more than 75% of the children reported no intake of these food groups.

Table S2. Model-fit statistics for latent class analysis

	Village A		Village B	
	**BIC**	**AIC**	**BIC**	**AIC**
Latent classes (dietary patterns) = 2	10909.29	10947.29	12695.89	12730.89
Latent classes (dietary patterns) = 3	10796.32	10856.32	12670.90	12725.90
Latent classes (dietary patterns) = 4	10789.43	10871.43	12667.80	12742.80
Latent classes (dietary patterns) = 5	10814.55	10918.55	12808.40	12903.40
Latent classes (dietary patterns) = 6	10926.30	11052.30	13040.49	13155.49
Latent classes (dietary patterns) = 7	11221.56	11369.56	13342.53	13477.53
